# Comparison of Deep Neural Networks for the Classification of Adventitious Lung Sounds

**DOI:** 10.3390/jcm14207427

**Published:** 2025-10-21

**Authors:** Said Polanco-Martagón, Yahir Hernández-Mier, Marco Aurelio Nuño-Maganda, José Hugo Barrón-Zambrano, Andrea Magadán-Salazar, César Alejandro Medellín-Vergara

**Affiliations:** 1 Intelligent Systems Department, Universidad Politécnica de Victoria, Ciudad Victoria 87138, Tamaulipas, Mexico; spolancom@upv.edu.mx (S.P.-M.); mnunom@upv.edu.mx (M.A.N.-M.); 1830077@upv.edu.mx (C.A.M.-V.); 2 Digital Systems Group, Electronics Department, Instituto Nacional de Astrofísica, Óptica y Electrónica, San Andrés Cholula 72840, Puebla, Mexico; jhbarron@inaoep.mx; 3 Computer Science Department, Tecnológico Nacional de México/CENIDET, Cuernavaca 62493, Morelos, Mexico; andrea.ms@cenidet.tecnm.mx

**Keywords:** deep learning, dual-stream neural network, adventitious sounds

## Abstract

**Background:** Automatic adventitious lung sound classification using deep learning is a promising strategy for objective respiratory disease screening. Evaluating model performance is challenging, particularly with imbalanced clinical datasets. This study compares CNN architectures and proposes a dual-stream classification approach. **Methods:** Using the public ICBHI 2017 dataset, we compared five pre-trained architectures: VGG16, VGG19, InceptionV3, MobileNetV2, and ResNet152V2. To mitigate class imbalance, we implemented pitch shifting, random shifting, and mixup data augmentation. We also developed and evaluated a novel VGGish-dual-stream network. The primary endpoint was the Average Score (AS), the arithmetic mean of Sensitivity and Specificity. **Results:** Among benchmarked models, ResNet152V2 achieved the highest AS (0.541), approaching the state-of-the-art range (0.56–0.58). This performance was characterised by a high Specificity (0.67) but low Sensitivity (0.41). Our proposed dual-stream network yielded a more balanced, albeit slightly lower, performance with an AS of 0.508. **Conclusions:** Standard CNN architectures like ResNet152V2 can achieve competitive classification performance but may exhibit a clinically significant bias towards high specificity at the expense of sensitivity. This trade-off poses a risk of missing pathological events (false negatives). To ensure clinical safety and utility, future work must prioritise strategies that explicitly improve model sensitivity.

## 1. Introduction

Chronic and acute respiratory disorders constitute a substantial global health burden, exerting considerable pressure on healthcare systems and accounting for millions of deaths annually [1]. While advanced diagnostic modalities, such as computed tomography, are essential for definitive diagnosis, pulmonary auscultation remains a fundamental component of frontline clinical evaluation due to its non-invasive nature, cost-effectiveness, and immediate utility [2]. However, the subjectivity of human interpretation constrains the diagnostic efficacy of auscultation. The nuanced and variable characteristics of adventitious sounds—such as crackles and wheezes—make their precise classification challenging even for experienced clinicians, which can lead to misdiagnosis and delayed treatment [3].

To address this limitation, computational lung sound analysis has evolved from traditional machine learning algorithms to deep learning, with Convolutional Neural Networks (CNNs) emerging as the predominant tool for automated classification [4]. Early and ongoing research has consistently demonstrated the viability of various CNN architectures, including VGG, ResNet, and Inception, for identifying pathological sounds from spectro-temporal representations such as spectrograms [5,6]. However, much of this work focuses on optimising performance metrics. This pursuit of a single high score often overlooks more clinically pertinent factors, such as model stability and robustness across heterogeneous patient data. High performance variability, or dispersion, is a critical barrier to establishing the clinical stability required for real-world adoption.

Several works have addressed the previously mentioned limitation, proposing more sophisticated architectures. Hybrid models, which combine CNNs with recurrent layers, multi-stream networks, and the integration of attention mechanisms, have all been proposed to enhance feature extraction and improve classification accuracy [7,8,9]. At the same time, clinical deployability has motivated the development of lightweight models designed for resource-constrained environments [10]. However, more complex models may increase computational cost, while lightweight models can sacrifice performance. Furthermore, across this architectural spectrum, the systematic evaluation of predictive consistency and reliability remains a secondary concern; therefore, researchers must focus on developing a model that is both accurate and consistent.

In this study, we propose a direct comparison of state-of-the-art architectures, which adheres to the official protocol of the ICBHI 2017 Scientific Challenge. We performed this comparison in two parts: first, we utilised a 60/40 training and testing data split without modification. Second, we evaluated our models using the Average Score (AS), calculated from Sensitivity and Specificity, which is the official challenge metric. This benchmarked approach provides a standardised framework for comparing several established CNN architectures. Following this, we introduce a novel dual-stream VGGish network, with the central hypothesis that this architecture will achieve a competitive AS, with lower dispersion compared to its single-stream counterparts. Proving this hypothesis would validate an architectural approach that prioritises reliability, a fundamental step in developing a clinically robust and stable automated auscultation system.

## 2. Methods

### 2.1. Study Design and Evaluation Framework

We designed this study as a comparative analysis of deep learning architectures for classifying adventitious lung sounds. To ensure direct comparability with existing literature, our methodology strictly adheres to the protocol of the ICBHI 2017 Scientific Challenge. We partition the dataset using the official 60% training and 40% testing split. We left out the test set and used it only for the final performance evaluation of the optimised models. We computed the AS as the arithmetic mean of Sensitivity (SE) and Specificity (SP). SE measures the model’s ability to correctly classify any adventitious sound (crackle, wheeze, or both), while SP measures the correct classification of normal respiratory cycles. Equations (1)–(3) define these metrics.

(1) $$SE=\frac{C_{t}+W_{t}+Bt}{C+W+B},$$

(2) $$SP=\frac{N_{t}}{N},$$

(3) $$AS=\frac{SE+SP}{2}.$$

### 2.2. Dataset

In this study, we utilised the publicly available ICBHI 2017 Challenge dataset [11]. This dataset contains 920 audio recordings from 126 patients, totalling 5.5 h of audio. The creators of the dataset acquired the recordings at sampling frequencies of 4 kHz, 10 kHz, and 44.1 kHz. Annotations provided within the dataset divide the recordings into 6898 distinct respiratory cycles, categorised as follows: 3642 normal, 1864 with crackles, 886 with wheezes, and 506 with both crackles and wheezes.

### 2.3. Signal Preprocessing and Feature Extraction

We applied a standardised preprocessing pipeline to all audio files. First, we use a third-order Butterworth bandpass filter (200–1800 Hz) to mitigate ambient noise. Second, we resampled all recordings to a uniform frequency of 4 kHz. Third, each respiratory cycle was padded or truncated to a fixed length of four seconds using zero-padding. Finally, Mel-spectrograms were generated from these processed audio clips to serve as the input features for all models. We computed the spectrograms using a Short-Time Fourier Transform (STFT) with a window size of 1024, a hop length of 512, and 128 Mel frequency bins.

### 2.4. Data Augmentation

To address the significant class imbalance in the training set and to improve model generalisation, we employed a series of data augmentation techniques. These were applied stochastically during the model training process. The specific methods and their application ratios are detailed in Table 1.

### 2.5. Experimental Models

We evaluated two categories of models: established benchmark architectures and a novel dual-stream network.

#### 2.5.1. Benchmark Architectures

We assessed the performance of five established CNN architectures: VGG16 [12], VGG19 [12], ResNet152V2 [13], InceptionV3 [14], and MobileNetV3-Large [15]. For all models, we employed transfer learning, initialising the networks with weights pre-trained on the ImageNet dataset. We replaced the final classification layers of each model with a new head consisting of a Global Average Pooling 2D layer followed by a dense layer with a softmax activation function for four-class classification.

#### 2.5.2. Proposed Dual-Stream VGGish Network

**Figure 1:**
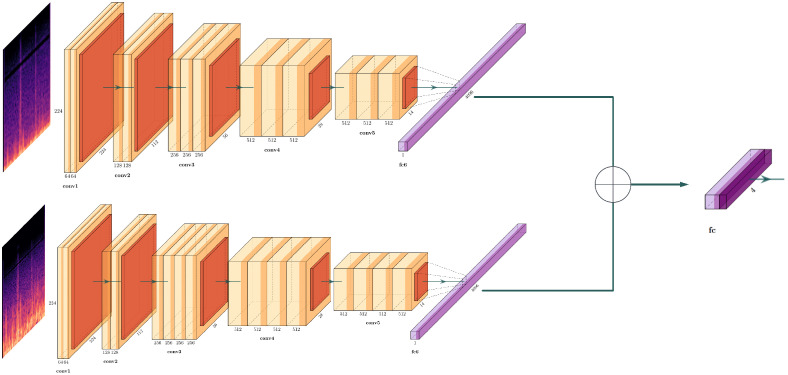
Proposed DS-CNN architecture. Convolutional layers are colored in light orange, while polling layers are colored in dark orange. Flattened feature layers are colored in light purple, while the dropout layer is colored in dark purple.

We developed a novel dual-stream architecture to assess its impact on performance stability (see Figure 1). The network comprises two parallel streams utilising VGG16 and VGG19 as feature extractors. The feature vectors extracted from the penultimate layer of each stream were concatenated and subsequently passed to a dense classification head. To leverage pre-trained knowledge while allowing for task-specific adaptation, we employed a differential fine-tuning strategy: the final convolutional block of each VGG backbone was unfrozen for training, while all earlier layers remained frozen.

### 2.6. Model Training and Evaluation

We trained all models using the TensorFlow Keras framework. For hyperparameter tuning, we internally partitioned 20% of the official training data as a validation set. We compared four different optimisers: Adam, SGD with momentum (0.9), Adamax, and RMSprop, with learning rates evaluated in the range of $1\times 10^{-6}$ to $1\times 10^{-4}$. We used the categorical cross-entropy loss function. To prevent overfitting, we employed an EarlyStopping callback monitoring the validation loss with a patience of 15 epochs.

Upon identifying the optimal hyperparameters for each architecture, we retrained the model on the entire training set (the full 60% of data). We then assessed the final, definitive performance a single time on the held-out test set.

### 2.7. Implementation Details

We conducted the experiments on a system equipped with an Intel i9-14900KF CPU (Santa Clara, CA, USA), an Nvidia GeForce RTX 4090 GPU (Santa Clara, CA, USA), and 32 GB of RAM. Key software packages included Python (v3.8.5), TensorFlow (v2.10), Scikit-learn (v1.3.2), and Librosa (v0.10.1). The complete source code for preprocessing, model implementation, and evaluation is publicly available at: https://github.com/CesarMVergara/Lung-Sounds-Classification (accessed on 15 October 2025).

## 3. Results

### 3.1. Overall Model Performance

We evaluated the performance of the five benchmark architectures and our proposed dual-stream network on the held-out test set, using the primary endpoint, the AS, alongside SE, SP, and per-class F1-scores for the optimal configuration of each model. We evaluated the six deep learning architectures using the metrics from the ICBHI 2017 challenge. The overall performance, based on a single training and evaluation run for each model’s optimal hyperparameter configuration, is summarised in Table 2.

### 3.2. Performance Analysis and Key Diagnostic Deficits

As shown in Table 2, the ResNet-152v2 architecture achieved the highest overall performance, yielding a top AS of 0.541. However, a critical and universal finding across all evaluated models was a pronounced trade-off between SE and SP. Every architecture demonstrated a systematic bias towards high specificity, indicating a greater reliability in confirming the absence of pathology (ruling out disease) than in detecting its presence (ruling in disease).

A second key finding pertains to the diagnostic capability per class. While most models showed moderate performance in detecting crackles, the classification of wheezes proved to be a significant and consistent challenge. This performance deficit was most extreme in the InceptionV3 model, which failed almost entirely on this task (F1-score = 0.070), suggesting a standard architectural limitation in capturing the distinct spectro-temporal features of these continuous, musical sounds.

### 3.3. Error Patterns and Clinical Behaviour of Top Models

**Figure 2:**
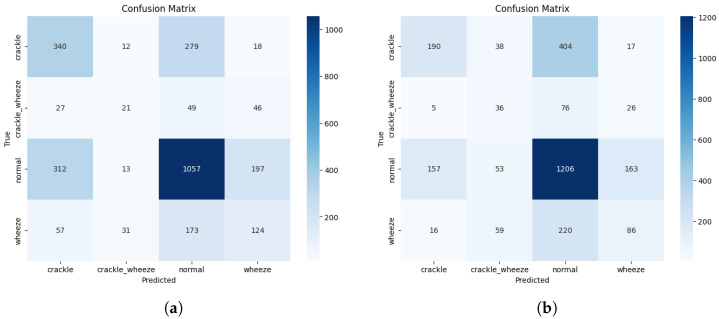
Confusion matrices for the best overall performing model (ResNet-152v2) and the proposed DS-CNN. Both illustrate a tendency to misclassify pathological events (crackle, wheeze, both) as normal. (**a**) ResNet-152v2 (Best AS). (**b**) DS-CNN (Highest Specificity).

The clinical behaviour behind these metrics is visualised in the confusion matrices for the top-performing and proposed models (Figure 2). The error analysis for the champion ResNet-152v2 model confirms that the predominant error type is the misclassification of adventitious sounds as ”normal”. This pattern translates to a high number of false negatives, which is a significant concern for any screening tool, as it implies missing pathological events.

Our proposed dual-stream CNN, while achieving a lower overall AS, exhibited the most extreme version of this behaviour. By attaining the highest Specificity (SP = 0.764) at the cost of the lowest Sensitivity (SE = 0.266), it behaved as a highly conservative model. Its tendency to classify ambiguous events as ‘normal’ makes it very effective at avoiding false alarms. Still, it consequently performs poorly at detecting disease, highlighting a critical trade-off that researchers must address for clinical utility.

## 4. Discussion

This study conducted a systematic comparison of deep learning architectures for the classification of adventitious lung sounds, leading to three principal findings. First, a fine-tuned ResNet-152v2 architecture achieved the highest overall performance. Second, all evaluated models exhibited a clinically significant trade-off, favouring high specificity at the cost of poor sensitivity. Third, our proposed dual-stream network, while not the top performer, showed preliminary signs of training stability, a key objective of our investigation.

### 4.1. Performance in the Context of the State-of-the-Art

Our top-performing model (ResNet-152v2, AS = 0.541) establishes a competitive benchmark, outperforming several published methodologies that use simpler or traditional models on the ICBHI 2017 dataset (Table 3). However, its performance falls short of the current state-of-the-art, where leading models achieve an AS between 0.56 and 0.58 [5,16]. This performance gap underscores that incremental gains in this field are no longer driven solely by standard architectural choices. Top-tier studies now distinguish themselves through advanced, non-architectural techniques such as domain-specific preprocessing (e.g., Black Region Clipping) and sophisticated regularisation methods (e.g., co-tuning frameworks).

Furthermore, the broader literature reveals a trend towards increasingly complex architectures to capture temporal features, such as hybrid CNN-LSTM models [7] and the integration of attention mechanisms [9]. While often effective, this complexity contrasts with a parallel drive towards lightweight models designed for real-world deployment on resource-constrained devices [10,24]. Our study, which focused on the performance of widely used foundational architectures, provides a critical reference point within this complex and sometimes contradictory research landscape.

### 4.2. Clinical Implications of the Sensitivity-Specificity Trade-Off

The most critical finding from a clinical perspective is the universal bias towards high specificity across all models. The low sensitivity of our champion model (SE = 0.412) translates to a high rate of false negatives, posing a significant patient safety risk by failing to detect the majority of pathological events. This finding is consistent with challenges reported elsewhere in the literature [25] and currently prevents the use of such models as stand-alone diagnostic or rule-out tools.

Conversely, the model’s high specificity suggests a different clinical utility. In a first-line care or triage setting, a high-specificity algorithm could function as an effective “normality filter”. Identifying healthy patients could help reduce unnecessary specialist referrals and diagnostic tests, thereby optimising resource allocation. The latter underscores an important principle: the clinical value of a diagnostic AI is defined not by a single performance score, but by the alignment of its specific error profile with a well-defined clinical need.

### 4.3. Limitations of the Study

It is important to contextualise our findings within the study’s limitations. A primary limitation is that we based the final model comparison on a single training run for each optimal configuration, not using a formal statistical analysis of performance variance. We adopted this approach due to the extensive computational burden associated with repeatedly training these deep neural network architectures. Consequently, we were unable to statistically prove our initial hypothesis regarding the superior stability of the dual-stream network, and this remains a critical direction for future work.

However, preliminary insights into model robustness can be inferred from the hyperparameter search phase (see Table A1, Table A2, Table A3, Table A4, Table A5 and Table A6). We observed that our proposed dual-stream CNN maintained a relatively consistent AS across different optimisers and a range of learning rates. The latter suggests a degree of robustness to hyperparameter selection, which contrasts with more sensitive models, such as ResNet152V2, whose performance was more dependent on a specific configuration. While not a substitute for repeated experiments with random seeds, this observation provides initial, indirect evidence that supports our architectural design choices, which aim to enhance stability, which is a goal often put as secondary in the related literature.

Further limitations include the use of the ICBHI 2017 dataset, whose modest cohort size may limit generalisability, and the controlled nature of the recordings, which may not capture the acoustic complexity of real-world clinical environments.

### 4.4. Future Directions

Based on these findings, future work should proceed along two main paths to achieve meaningful and safe clinical deployment. First, to rigorously test the stability hypothesis, a comprehensive evaluation involving repeated, seeded training runs is of primary importance to quantify the performance variance and provide confidence intervals for all reported metrics. Second, to enhance clinical trust and interpretability, the integration of explainability techniques, such as Grad-CAM, is necessary to visualise the spectrogram features that drive model predictions.

## 5. Conclusions

This study demonstrates that while standard deep learning architectures can achieve competitive performance in classifying adventitious lung sounds, their utility is universally constrained by a critical trade-off: a high specificity achieved at the cost of a clinically concerning low sensitivity. This systematic bias towards misclassifying pathological events as normal suggests that the search for a single, optimised performance score, commonly found in the machine learning field, could limit its safe clinical application.

Consequently, the principal contribution of this work is not the identification of a superior architecture, but the characterisation of this performance profile. Future progress must go from solely maximizing accuracy to explicitly engineering for specific clinical needs. Our exploration of a dual-stream network, designed to enhance predictive stability, represents an initial step in this direction, prioritising reliability over a singular performance metric.

Ultimately, architectural innovation alone does not translate automated auscultation from a research concept to a stable clinical tool. It will require a paradigm shift towards rigorous statistical validation, a focus on model interpretability, and the careful alignment of an algorithm’s specific error profile with a well-defined clinical context.

## Figures and Tables

**Table 1:** Data augmentation technique applied.

Technique	Description	Ratio
Pitch shift	Pitch shifting alters the fundamental frequency of an audio signal without affecting its duration.	20% random
Random shift	Random time-shifting shifts the audio signal along the time axis.	20% random
Volume Adjust	Volume adjustment involves altering the amplitude of an audio sample	20% random
Time streching	Time stretching modifies the duration of an audio signal while preserving its pitch.	20% random
Mixup	Create new training samples that interpolate between existing examples.	10% random “normal-crackle”
		10% random “normal-wheeze”
		10% random “crackle-wheeze”

**Table 2:** Comparative performance of all evaluated architectures on the test set.

Model	Optimizer	LR	AS	SE	SP	F1-Normal	F1-Crackle	F1-Wheeze	F1-Both
ResNet-152v2	Adam	$5.0\times 10^{-5}$	0.541	0.412	0.669	0.674	0.491	0.191	0.322
VGG16	Adam	$1.0\times 10^{-5}$	0.531	0.316	0.746	0.721	0.464	0.220	0.260
VGG19	RMSprop	$5.0\times 10^{-5}$	0.528	0.370	0.685	0.672	0.418	0.242	0.361
Dual-Stream CNN	Adam	$5.0\times 10^{-6}$	0.515	0.266	0.764	0.692	0.374	0.219	0.256
InceptionV3	Adam	$1.0\times 10^{-4}$	0.498	0.314	0.682	0.664	0.432	0.070	0.264
MobileNetV3L	RMSprop	$1.0\times 10^{-4}$	0.476	0.347	0.605	0.629	0.411	0.211	0.208

**Table 3:** Comparison with the state of the art.

Ref.	ML Method	Input	Sensitivity	Specificity	AS
[17]	ResNet-18 + SE Block + Spatial Attention Block	STFT spectrograms	17.84	81.25	49.55
[16]	CNN	Black Region Clipping from Mel Spectrograms	39.0	71.4	55.2
	CNN + CBA + BRC	Black Region Clipping from Mel Spectrograms	39.6	71.8	55.7
	CNN + CBA + BRC + FT	Black Region Clipping from Mel Spectrograms	40.1	72.3	56.2
[18]	LungRN + NL	STFT spectrograms	41.32	63.20	52.26
[5]	ResNet50 + co-tuning + Stochastic Normalization	log-mel spectrograms	37.24	79.34	58.29
[19]	CNN-MoE	Gamma Spectrogram	0.26	0.68	0.47
[20]	Bi-ResNet	Wavelet analysis + STFT	31.12	69.20	50.16
[21]	HMM	MFCC	0.4232	0.5669	39.37
	HMM	MFCC	0.4890	0.7780	49.86
[22]	Hidden Markov Models	temporal and spectral characteristics	N/A	N/A	39.37
[23]	Blnet	STFT + WT	42.63	61.33	51.98
Our results					
Our Results	ResNet152V2	Mel-Specrogram	41.20	66.94	54.07
	VGG16	Mel-Specrogram	31.62	74.60	53.11
	VGG19	Mel-Specrogram	37.04	68.52	52.78
	Propposed DS-CNN	Mel-Specrogram	26.59	76.37	51.48
	InceptionV3	Mel-Specrogram	31.43	68.20	49.82
	MobileNetV3 Large	Mel-Specrogram	34.66	60.54	47.60

**Figure A1:**
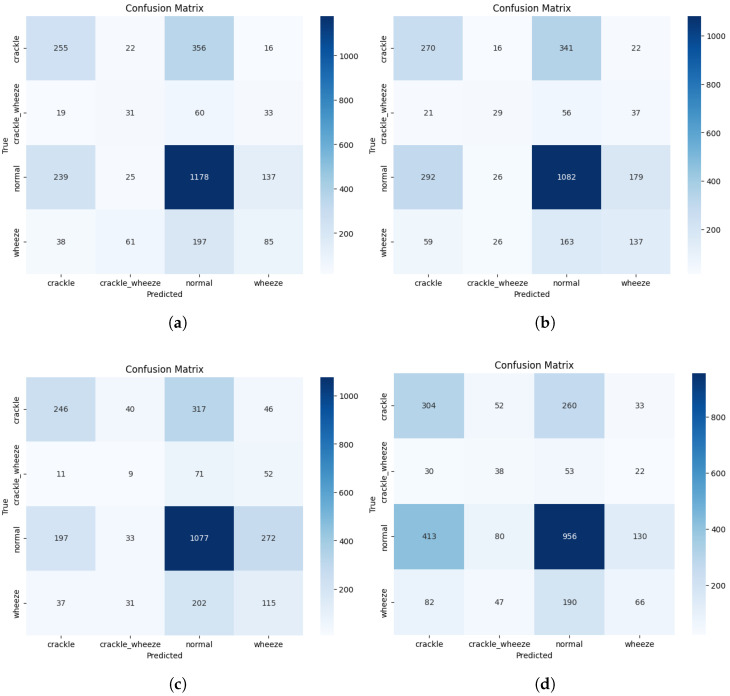
(**a**) Confusion matrix for the optimal VGG16 model (Adam, LR 1.0 × 10^−5^). (**b**) Confusion matrix for the optimal VGG19 model (RMSprop, LR 5.0 × 10^−5^). (**c**) Confusion matrix for the optimal InceptionV3 model (Adam, LR 1.0 × 10^−4^). (**d**) Confusion matrix for the optimal MobileNetV3L model (RMSprop, LR 1.0 × 10^−4^).

## Data Availability

Restrictions apply to the availability of these data. Data were obtained from ICBHI 2017 challenge and are available https://doi.org/10.7910/DVN/HT6PKI with the permission of Int. Conf. on Biomedical Health Informatics - ICBHI 2017. [ICBHI 2017 challenge] [https://doi.org/10.7910/DVN/HT6PKI].

## Appendix A

This supplementary material provides information to the main manuscript, “Comparison of deep neural networks for the classification of adventitious lung sounds”. It includes the detailed results of the hyperparameter search for all evaluated models and the complete set of confusion matrices.

### Appendix A.1. Detailed Hyperparameter Search Results

The following tables (Table A1, Table A2, Table A3, Table A4, Table A5 and Table A6) present the complete performance metrics for every hyperparameter configuration evaluated in this study. The optimal configuration for each model, which is reported in the main manuscript’s Table 2, is highlighted in bold for clarity.

#### Appendix A.1.1. VGG16 Hyperparameter Search Results

Table A1 details the performance metrics for the VGG16 architecture across all evaluated optimisers and learning rates. The best performance was achieved with the Adam optimiser at a learning rate of $1.0\times 10^{-5}$.

**Table A1:** VGG16 results.

Optimizer	Learning Rate	Accuracy	Precision	Recall	F1 Score	Sensitivity	Specificity	AS
Adam	1.0000 × 10^−6^	5.3525 × 10^−1^	5.5200 × 10^−1^	5.3525 × 10^−1^	5.4269 × 10^−1^	3.9471 × 10^−1^	6.3965 × 10^−1^	5.1718 × 10^−1^
	5.0000 × 10^−6^	5.5596 × 10^−1^	5.3994 × 10^−1^	5.5596 × 10^−1^	5.4518 × 10^−1^	3.2225 × 10^−1^	7.2958 × 10^−1^	5.2591 × 10^−1^
	1.0000 × 10^−5^	5.6286 × 10^−1^	5.4154 × 10^−1^	5.6286 × 10^−1^	5.4887 × 10^−1^	3.1628 × 10^−1^	7.4604 × 10^−1^	5.3116 × 10^−1^
	5.0000 × 10^−5^	5.5451 × 10^−1^	5.4665 × 10^−1^	5.5451 × 10^−1^	5.4864 × 10^−1^	3.4101 × 10^−1^	7.1311 × 10^−1^	5.2706 × 10^−1^
	1.0000 × 10^−4^	5.5015 × 10^−1^	5.3169 × 10^−1^	5.5015 × 10^−1^	5.3865 × 10^−1^	3.0776 × 10^−1^	7.3021 × 10^−1^	5.1898 × 10^−1^
SGD	1.0000 × 10^−6^	4.1533 × 10^−1^	4.9613 × 10^−1^	4.1533 × 10^−1^	4.4743 × 10^−1^	2.6428 × 10^−1^	5.2755 × 10^−1^	3.9591 × 10^−1^
	5.0000 × 10^−6^	4.6512 × 10^−1^	4.4143 × 10^−1^	4.6512 × 10^−1^	4.2971 × 10^−1^	8.0989 × 10^−2^	7.5047 × 10^−1^	4.1573 × 10^−1^
	1.0000 × 10^−5^	4.0675 × 10^−1^	4.8635 × 10^−1^	4.0675 × 10^−1^	4.3261 × 10^−1^	3.0501 × 10^−1^	4.8258 × 10^−1^	3.9380 × 10^−1^
	5.0000 × 10^−5^	4.7279 × 10^−1^	5.5738 × 10^−1^	4.7279 × 10^−1^	4.8956 × 10^−1^	4.6219 × 10^−1^	4.8068 × 10^−1^	4.7144 × 10^−1^
	1.0000 × 10^−4^	5.1052 × 10^−1^	5.4678 × 10^−1^	5.1052 × 10^−1^	5.2481 × 10^−1^	4.1801 × 10^−1^	5.7948 × 10^−1^	4.9875 × 10^−1^
Adamax	1.0000 × 10^−6^	5.0981 × 10^−1^	5.3254 × 10^−1^	5.0981 × 10^−1^	5.1893 × 10^−1^	3.7852 × 10^−1^	6.0735 × 10^−1^	4.9293 × 10^−1^
	5.0000 × 10^−6^	5.3307 × 10^−1^	5.3770 × 10^−1^	5.3307 × 10^−1^	5.3495 × 10^−1^	3.7340 × 10^−1^	6.5168 × 10^−1^	5.1254 × 10^−1^
	1.0000 × 10^−5^	5.4862 × 10^−1^	5.3326 × 10^−1^	5.4862 × 10^−1^	5.3803 × 10^−1^	3.4410 × 10^−1^	7.0108 × 10^−1^	5.2259 × 10^−1^
	5.0000 × 10^−5^	5.5878 × 10^−1^	5.3389 × 10^−1^	5.5878 × 10^−1^	5.3834 × 10^−1^	2.7867 × 10^−1^	7.6757 × 10^−1^	5.2312 × 10^−1^
	1.0000 × 10^−4^	5.5443 × 10^−1^	5.3621 × 10^−1^	5.5443 × 10^−1^	5.4307 × 10^−1^	3.3305 × 10^−1^	7.1944 × 10^−1^	5.2625 × 10^−1^
RMSprop	1.0000 × 10^−6^	5.1415 × 10^−1^	5.2238 × 10^−1^	5.1415 × 10^−1^	5.1649 × 10^−1^	3.3390 × 10^−1^	6.4851 × 10^−1^	4.9121 × 10^−1^
	5.0000 × 10^−6^	4.7279 × 10^−1^	5.0541 × 10^−1^	4.7279 × 10^−1^	4.8383 × 10^−1^	3.9762 × 10^−1^	5.2882 × 10^−1^	4.6322 × 10^−1^
	1.0000 × 10^−5^	4.6081 × 10^−1^	4.9144 × 10^−1^	4.6081 × 10^−1^	4.7290 × 10^−1^	3.1351 × 10^−1^	5.7061 × 10^−1^	4.4206 × 10^−1^
	5.0000 × 10^−5^	5.2504 × 10^−1^	5.5282 × 10^−1^	5.2504 × 10^−1^	5.3373 × 10^−1^	4.2906 × 10^−1^	5.9658 × 10^−1^	5.1282 × 10^−1^
	1.0000 × 10^−4^	5.3628 × 10^−1^	5.1257 × 10^−1^	5.3628 × 10^−1^	5.1356 × 10^−1^	2.5913 × 10^−1^	7.4288 × 10^−1^	5.0100 × 10^−1^

#### Appendix A.1.2. VGG19 Hyperparameter Search Results

Table A2 details the performance metrics for the VGG19 architecture across all evaluated optimisers and learning rates. The best performance was achieved with the RMSprop optimiser at a learning rate of 5.0 × 10^−5^.

**Table A2:** VGG19 results.

Optimizer	Learning Rate	Accuracy	Precision	Recall	F1 Score	Sensitivity	Specificity	AS
Adam	1.0000 × 10^−6^	4.7460 × 10^−1^	5.1438 × 10^−1^	4.7460 × 10^−1^	4.8965 × 10^−1^	3.3390 × 10^−1^	5.7948 × 10^−1^	4.5669 × 10^−1^
	5.0000 × 10^−6^	4.9710 × 10^−1^	5.2736 × 10^−1^	4.9710 × 10^−1^	5.0785 × 10^−1^	4.1546 × 10^−1^	5.5795 × 10^−1^	4.8671 × 10^−1^
	1.0000 × 10^−5^	5.0000 × 10^−1^	5.2950 × 10^−1^	5.0000 × 10^−1^	5.1129 × 10^−1^	3.7893 × 10^−1^	5.9025 × 10^−1^	4.8459 × 10^−1^
	5.0000 × 10^−5^	5.1161 × 10^−1^	5.0075 × 10^−1^	5.1161 × 10^−1^	5.0493 × 10^−1^	2.9822 × 10^−1^	6.7068 × 10^−1^	4.8445 × 10^−1^
	1.0000 × 10^−4^	4.6662 × 10^−1^	4.8222 × 10^−1^	4.6662 × 10^−1^	4.7142 × 10^−1^	2.6083 × 10^−1^	6.2001 × 10^−1^	4.4042 × 10^−1^
SGD	1.0000 × 10^−6^	3.0406 × 10^−1^	4.6163 × 10^−1^	3.0406 × 10^−1^	3.4549 × 10^−1^	2.1155 × 10^−1^	3.7302 × 10^−1^	2.9229 × 10^−1^
	5.0000 × 10^−6^	4.5610 × 10^−1^	5.0038 × 10^−1^	4.5610 × 10^−1^	4.7248 × 10^−1^	3.4834 × 10^−1^	5.3642 × 10^−1^	4.4238 × 10^−1^
	1.0000 × 10^−5^	4.5501 × 10^−1^	5.1179 × 10^−1^	4.5501 × 10^−1^	4.7550 × 10^−1^	3.6194 × 10^−1^	5.2438 × 10^−1^	4.4316 × 10^−1^
	5.0000 × 10^−5^	4.6335 × 10^−1^	5.2275 × 10^−1^	4.6335 × 10^−1^	4.7863 × 10^−1^	4.4605 × 10^−1^	4.7625 × 10^−1^	4.6115 × 10^−1^
	1.0000 × 10^−4^	5.0109 × 10^−1^	5.2580 × 10^−1^	5.0109 × 10^−1^	5.0957 × 10^−1^	3.5004 × 10^−1^	6.1368 × 10^−1^	4.8186 × 10^−1^
Adamax	1.0000 × 10^−6^	4.6480 × 10^−1^	5.0736 × 10^−1^	4.6480 × 10^−1^	4.8046 × 10^−1^	3.4070 × 10^−1^	5.5731 × 10^−1^	4.4901 × 10^−1^
	5.0000 × 10^−6^	4.8984 × 10^−1^	5.0930 × 10^−1^	4.8984 × 10^−1^	4.9440 × 10^−1^	3.0926 × 10^−1^	6.2445 × 10^−1^	4.6685 × 10^−1^
	1.0000 × 10^−5^	4.7823 × 10^−1^	4.9803 × 10^−1^	4.7823 × 10^−1^	4.8488 × 10^−1^	3.1096 × 10^−1^	6.0291 × 10^−1^	4.5694 × 10^−1^
	5.0000 × 10^−5^	5.0073 × 10^−1^	5.0765 × 10^−1^	5.0073 × 10^−1^	5.0375 × 10^−1^	3.5429 × 10^−1^	6.0988 × 10^−1^	4.8209 × 10^−1^
	1.0000 × 10^−4^	4.8948 × 10^−1^	4.8124 × 10^−1^	4.8948 × 10^−1^	4.8438 × 10^−1^	2.9737 × 10^−1^	6.3268 × 10^−1^	4.6502 × 10^−1^
RMSprop	1.0000 × 10^−6^	4.8766 × 10^−1^	4.9777 × 10^−1^	4.8766 × 10^−1^	4.9070 × 10^−1^	3.1521 × 10^−1^	6.1621 × 10^−1^	4.6571 × 10^−1^
	5.0000 × 10^−6^	5.1851 × 10^−1^	5.2591 × 10^−1^	5.1851 × 10^−1^	5.2070 × 10^−1^	3.8488 × 10^−1^	6.1811 × 10^−1^	5.0149 × 10^−1^
	1.0000 × 10^−5^	4.6009 × 10^−1^	5.0493 × 10^−1^	4.6009 × 10^−1^	4.7315 × 10^−1^	4.2226 × 10^−1^	4.8828 × 10^−1^	4.5527 × 10^−1^
	5.0000 × 10^−5^	5.5080 × 10^−1^	5.4312 × 10^−1^	5.5080 × 10^−1^	5.4632 × 10^−1^	3.7043 × 10^−1^	6.8524 × 10^−1^	5.2784 × 10^−1^
	1.0000 × 10^−4^	5.4173 × 10^−1^	5.2417 × 10^−1^	5.4173 × 10^−1^	5.2784 × 10^−1^	2.9567 × 10^−1^	7.2514 × 10^−1^	5.1040 × 10^−1^

#### Appendix A.1.3. ResNet-152v2 Hyperparameter Search Results

Table A3 details the performance metrics for the ResNet-152v2 architecture across all evaluated optimisers and learning rates. The best performance was achieved with the Adam optimiser at a learning rate of 5.0 × 10^−5^.

**Table A3:** ResNet 152 Ver. 2. results.

Optimizer	Learning Rate	Accuracy	Precision	Recall	F1 Score	Sensitivity	Specificity	AS
Adam	1.0000 × 10^−6^	4.5682 × 10^−1^	4.9892 × 10^−1^	4.5682 × 10^−1^	4.7248 × 10^−1^	3.5854 × 10^−1^	5.3008 × 10^−1^	4.4431 × 10^−1^
	5.0000 × 10^−6^	5.0726 × 10^−1^	5.1384 × 10^−1^	5.0726 × 10^−1^	5.0850 × 10^−1^	3.3985 × 10^−1^	6.3205 × 10^−1^	4.8595 × 10^−1^
	1.0000 × 10^−5^	5.1851 × 10^−1^	5.1149 × 10^−1^	5.1851 × 10^−1^	5.1422 × 10^−1^	3.1861 × 10^−1^	6.6751 × 10^−1^	4.9306 × 10^−1^
	5.0000 × 10^−5^	5.5951 × 10^−1^	5.5662 × 10^−1^	5.5951 × 10^−1^	5.5661 × 10^−1^	4.1206 × 10^−1^	6.6941 × 10^−1^	5.4074 × 10^−1^
	1.0000 × 10^−4^	5.5951 × 10^−1^	5.5205 × 10^−1^	5.5951 × 10^−1^	5.5502 × 10^−1^	3.6958 × 10^−1^	7.0108 × 10^−1^	5.3533 × 10^−1^
SGD	1.0000 × 10^−6^	2.7540 × 10^−1^	4.1012 × 10^−1^	2.7540 × 10^−1^	3.1078 × 10^−1^	2.5828 × 10^−1^	2.8816 × 10^−1^	2.7322 × 10^−1^
	5.0000 × 10^−6^	3.7083 × 10^−1^	4.8208 × 10^−1^	3.7083 × 10^−1^	4.0685 × 10^−1^	2.9737 × 10^−1^	4.2559 × 10^−1^	3.6148 × 10^−1^
	1.0000 × 10^−5^	4.1618 × 10^−1^	4.9889 × 10^−1^	4.1618 × 10^−1^	4.4427 × 10^−1^	3.4664 × 10^−1^	4.6802 × 10^−1^	4.0733 × 10^−1^
	5.0000 × 10^−5^	4.5428 × 10^−1^	4.9868 × 10^−1^	4.5428 × 10^−1^	4.6981 × 10^−1^	3.6619 × 10^−1^	5.1995 × 10^−1^	4.4307 × 10^−1^
	1.0000 × 10^−4^	4.5537 × 10^−1^	5.0861 × 10^−1^	4.5537 × 10^−1^	4.7256 × 10^−1^	3.8403 × 10^−1^	5.0855 × 10^−1^	4.4629 × 10^−1^
Adamax	1.0000 × 10^−6^	4.3578 × 10^−1^	4.8617 × 10^−1^	4.3578 × 10^−1^	4.5358 × 10^−1^	3.5684 × 10^−1^	4.9462 × 10^−1^	4.2573 × 10^−1^
	5.0000 × 10^−6^	4.4993 × 10^−1^	4.8864 × 10^−1^	4.4993 × 10^−1^	4.6473 × 10^−1^	3.4664 × 10^−1^	5.2692 × 10^−1^	4.3678 × 10^−1^
	1.0000 × 10^−5^	4.6299 × 10^−1^	4.8579 × 10^−1^	4.6299 × 10^−1^	4.7280 × 10^−1^	3.1691 × 10^−1^	5.7188 × 10^−1^	4.4439 × 10^−1^
	5.0000 × 10^−5^	4.4739 × 10^−1^	5.1449 × 10^−1^	4.4739 × 10^−1^	4.6801 × 10^−1^	4.1716 × 10^−1^	4.6992 × 10^−1^	4.4354 × 10^−1^
	1.0000 × 10^−4^	4.9311 × 10^−1^	5.4088 × 10^−1^	4.9311 × 10^−1^	5.0160 × 10^−1^	4.8768 × 10^−1^	4.9715 × 10^−1^	4.9242 × 10^−1^
RMSprop	1.0000 × 10^−6^	4.6081 × 10^−1^	5.0289 × 10^−1^	4.6081 × 10^−1^	4.7606 × 10^−1^	3.6873 × 10^−1^	5.2945 × 10^−1^	4.4909 × 10^−1^
	5.0000 × 10^−6^	4.3360 × 10^−1^	4.9977 × 10^−1^	4.3360 × 10^−1^	4.5638 × 10^−1^	3.6449 × 10^−1^	4.8512 × 10^−1^	4.2480 × 10^−1^
	1.0000 × 10^−5^	5.2177 × 10^−1^	5.0973 × 10^−1^	5.2177 × 10^−1^	5.1434 × 10^−1^	3.0501 × 10^−1^	6.8334 × 10^−1^	4.9418 × 10^−1^
	5.0000 × 10^−5^	4.8258 × 10^−1^	5.3932 × 10^−1^	4.8258 × 10^−1^	4.9803 × 10^−1^	4.5879 × 10^−1^	5.0032 × 10^−1^	4.7956 × 10^−1^
	1.0000 × 10^−4^	5.2576 × 10^−1^	5.2796 × 10^−1^	5.2576 × 10^−1^	5.2072 × 10^−1^	4.0442 × 10^−1^	6.1621 × 10^−1^	5.1032 × 10^−1^

#### Appendix A.1.4. InceptionV3 Hyperparameter Search Results

Table A4 details the performance metrics for the InceptionV3 architecture across all evaluated optimisers and learning rates. The best performance was achieved with the Adam optimiser at a learning rate of 1.0 × 10^−4^.

**Table A4:** Inception Ver. 3. results.

Optimizer	Learning Rate	Accuracy	Precision	Recall	F1 Score	Sensitivity	Specificity	AS
Adam	1.0000 × 10^−6^	4.3142 × 10^−1^	4.8412 × 10^−1^	4.3142 × 10^−1^	4.5106 × 10^−1^	3.2116 × 10^−1^	5.1362 × 10^−1^	4.1739 × 10^−1^
	5.0000 × 10^−6^	4.3251 × 10^−1^	4.8467 × 10^−1^	4.3251 × 10^−1^	4.5266 × 10^−1^	2.9992 × 10^−1^	5.3135 × 10^−1^	4.1563 × 10^−1^
	1.0000 × 10^−5^	4.4013 × 10^−1^	4.7252 × 10^−1^	4.4013 × 10^−1^	4.5352 × 10^−1^	2.9312 × 10^−1^	5.4972 × 10^−1^	4.2142 × 10^−1^
	5.0000 × 10^−5^	5.0218 × 10^−1^	4.9777 × 10^−1^	5.0218 × 10^−1^	4.9601 × 10^−1^	2.5913 × 10^−1^	6.8334 × 10^−1^	4.7124 × 10^−1^
	1.0000 × 10^−4^	5.2504 × 10^−1^	5.2539 × 10^−1^	5.2504 × 10^−1^	5.2240 × 10^−1^	3.1436 × 10^−1^	6.8208 × 10^−1^	4.9822 × 10^−1^
SGD	1.0000 × 10^−6^	4.0348 × 10^−1^	3.9958 × 10^−1^	4.0348 × 10^−1^	3.8984 × 10^−1^	1.1045 × 10^−1^	6.2191 × 10^−1^	3.6618 × 10^−1^
	5.0000 × 10^−6^	3.7845 × 10^−1^	4.0297 × 10^−1^	3.7845 × 10^−1^	3.8006 × 10^−1^	1.2999 × 10^−1^	5.6365 × 10^−1^	3.4682 × 10^−1^
	1.0000 × 10^−5^	3.7663 × 10^−1^	4.8316 × 10^−1^	3.7663 × 10^−1^	4.1074 × 10^−1^	3.1011 × 10^−1^	4.2622 × 10^−1^	3.6816 × 10^−1^
	5.0000 × 10^−5^	4.2017 × 10^−1^	4.8548 × 10^−1^	4.2017 × 10^−1^	4.4246 × 10^−1^	3.3050 × 10^−1^	4.8702 × 10^−1^	4.0876 × 10^−1^
	1.0000 × 10^−4^	4.4194 × 10^−1^	4.9237 × 10^−1^	4.4194 × 10^−1^	4.6119 × 10^−1^	3.2370 × 10^−1^	5.3008 × 10^−1^	4.2689 × 10^−1^
Adamax	1.0000 × 10^−6^	4.2997 × 10^−1^	4.8317 × 10^−1^	4.2997 × 10^−1^	4.4929 × 10^−1^	3.2540 × 10^−1^	5.0792 × 10^−1^	4.1666 × 10^−1^
	5.0000 × 10^−6^	4.3541 × 10^−1^	4.8750 × 10^−1^	4.3541 × 10^−1^	4.5463 × 10^−1^	3.3135 × 10^−1^	5.1298 × 10^−1^	4.2217 × 10^−1^
	1.0000 × 10^−5^	4.3469 × 10^−1^	4.8510 × 10^−1^	4.3469 × 10^−1^	4.5390 × 10^−1^	3.0586 × 10^−1^	5.3072 × 10^−1^	4.1829 × 10^−1^
	5.0000 × 10^−5^	4.6118 × 10^−1^	4.8240 × 10^−1^	4.6118 × 10^−1^	4.6974 × 10^−1^	2.9142 × 10^−1^	5.8771 × 10^−1^	4.3957 × 10^−1^
	1.0000 × 10^−4^	4.7388 × 10^−1^	4.9497 × 10^−1^	4.7388 × 10^−1^	4.8179 × 10^−1^	3.3560 × 10^−1^	5.7695 × 10^−1^	4.5627 × 10^−1^
RMSprop	1.0000 × 10^−6^	4.3687 × 10^−1^	4.8795 × 10^−1^	4.3687 × 10^−1^	4.5550 × 10^−1^	3.3050 × 10^−1^	5.1615 × 10^−1^	4.2333 × 10^−1^
	5.0000 × 10^−6^	4.4086 × 10^−1^	4.8564 × 10^−1^	4.4086 × 10^−1^	4.5731 × 10^−1^	3.2880 × 10^−1^	5.2438 × 10^−1^	4.2659 × 10^−1^
	1.0000 × 10^−5^	4.7134 × 10^−1^	4.7446 × 10^−1^	4.7134 × 10^−1^	4.6810 × 10^−1^	2.8717 × 10^−1^	6.0861 × 10^−1^	4.4789 × 10^−1^
	5.0000 × 10^−5^	4.9673 × 10^−1^	5.0369 × 10^−1^	4.9673 × 10^−1^	4.9184 × 10^−1^	3.5599 × 10^−1^	6.0165 × 10^−1^	4.7882 × 10^−1^
	1.0000 × 10^−4^	4.8476 × 10^−1^	5.3610 × 10^−1^	4.8476 × 10^−1^	4.8922 × 10^−1^	4.6304 × 10^−1^	5.0095 × 10^−1^	4.8200 × 10^−1^

#### Appendix A.1.5. MobileNetV3-Large Hyperparameter Search Results

Table A5 details the performance metrics for the MobileNetV3-Large architecture across all evaluated optimisers and learning rates. The best performance was achieved with the RMSprop optimiser at a learning rate of 1.0 × 10^−4^.

**Table A5:** MobileNet Ver. 3 results.

Optimizer	Learning Rate	Accuracy	Precision	Recall	F1 Score	Sensitivity	Specificity	AS
Adam	1.0000 × 10^−6^	4.8367 × 10^−1^	5.0495 × 10^−1^	4.8367 × 10^−1^	4.9297 × 10^−1^	3.2965 × 10^−1^	5.9848 × 10^−1^	4.6407 × 10^−1^
	5.0000 × 10^−6^	4.5900 × 10^−1^	4.8548 × 10^−1^	4.5900 × 10^−1^	4.7001 × 10^−1^	3.1351 × 10^−1^	5.6745 × 10^−1^	4.4048 × 10^−1^
	1.0000 × 10^−5^	4.3070 × 10^−1^	4.8452 × 10^−1^	4.3070 × 10^−1^	4.5081 × 10^−1^	3.2710 × 10^−1^	5.0792 × 10^−1^	4.1751 × 10^−1^
	5.0000 × 10^−5^	4.5174 × 10^−1^	5.1933 × 10^−1^	4.5174 × 10^−1^	4.7154 × 10^−1^	3.9507 × 10^−1^	4.9398 × 10^−1^	4.4453 × 10^−1^
	1.0000 × 10^−4^	4.1364 × 10^−1^	4.9162 × 10^−1^	4.1364 × 10^−1^	4.3592 × 10^−1^	3.9677 × 10^−1^	4.2622 × 10^−1^	4.1150 × 10^−1^
SGD	1.0000 × 10^−6^	3.5740 × 10^−1^	4.5936 × 10^−1^	3.5740 × 10^−1^	3.9479 × 10^−1^	2.2260 × 10^−1^	4.5788 × 10^−1^	3.4024 × 10^−1^
	5.0000 × 10^−6^	4.0893 × 10^−1^	4.7948 × 10^−1^	4.0893 × 10^−1^	4.3612 × 10^−1^	2.7613 × 10^−1^	5.0792 × 10^−1^	3.9202 × 10^−1^
	1.0000 × 10^−5^	4.2707 × 10^−1^	4.7673 × 10^−1^	4.2707 × 10^−1^	4.4812 × 10^−1^	2.6253 × 10^−1^	5.4972 × 10^−1^	4.0612 × 10^−1^
	5.0000 × 10^−5^	4.7896 × 10^−1^	4.9378 × 10^−1^	4.7896 × 10^−1^	4.8547 × 10^−1^	3.0416 × 10^−1^	6.0925 × 10^−1^	4.5670 × 10^−1^
	1.0000 × 10^−4^	4.8803 × 10^−1^	5.0277 × 10^−1^	4.8803 × 10^−1^	4.9464 × 10^−1^	3.1946 × 10^−1^	6.1368 × 10^−1^	4.6657 × 10^−1^
Adamax	1.0000 × 10^−6^	4.7134 × 10^−1^	4.9028 × 10^−1^	4.7134 × 10^−1^	4.7990 × 10^−1^	2.9992 × 10^−1^	5.9911 × 10^−1^	4.4951 × 10^−1^
	5.0000 × 10^−6^	4.8440 × 10^−1^	5.1205 × 10^−1^	4.8440 × 10^−1^	4.9582 × 10^−1^	3.3985 × 10^−1^	5.9215 × 10^−1^	4.6600 × 10^−1^
	1.0000 × 10^−5^	4.5319 × 10^−1^	5.0370 × 10^−1^	4.5319 × 10^−1^	4.7177 × 10^−1^	3.5089 × 10^−1^	5.2945 × 10^−1^	4.4017 × 10^−1^
	5.0000 × 10^−5^	4.7750 × 10^−1^	4.9463 × 10^−1^	4.7750 × 10^−1^	4.8464 × 10^−1^	3.2201 × 10^−1^	5.9341 × 10^−1^	4.5771 × 10^−1^
	1.0000 × 10^−4^	4.6626 × 10^−1^	4.9861 × 10^−1^	4.6626 × 10^−1^	4.7893 × 10^−1^	3.4325 × 10^−1^	5.5795 × 10^−1^	4.5060 × 10^−1^
RMSprop	1.0000 × 10^−6^	4.8367 × 10^−1^	5.0343 × 10^−1^	4.8367 × 10^−1^	4.9233 × 10^−1^	3.2710 × 10^−1^	6.0038 × 10^−1^	4.6374 × 10^−1^
	5.0000 × 10^−6^	4.7351 × 10^−1^	4.9574 × 10^−1^	4.7351 × 10^−1^	4.8039 × 10^−1^	3.5514 × 10^−1^	5.6175 × 10^−1^	4.5844 × 10^−1^
	1.0000 × 10^−5^	4.7896 × 10^−1^	4.9615 × 10^−1^	4.7896 × 10^−1^	4.8362 × 10^−1^	3.4155 × 10^−1^	5.8138 × 10^−1^	4.6146 × 10^−1^
	5.0000 × 10^−5^	4.2598 × 10^−1^	4.9908 × 10^−1^	4.2598 × 10^−1^	4.2910 × 10^−1^	4.3925 × 10^−1^	4.1609 × 10^−1^	4.2767 × 10^−1^
	1.0000 × 10^−4^	4.9492 × 10^−1^	5.0758 × 10^−1^	4.9492 × 10^−1^	4.9740 × 10^−1^	3.4664 × 10^−1^	6.0545 × 10^−1^	4.7605 × 10^−1^

#### Appendix A.1.6. Dual-Stream CNN Hyperparameter Search Results

Table A6 details the performance metrics for the proposed dual-stream CNN architecture across all evaluated optimisers and learning rates. The best performance was achieved with the Adam optimiser at a learning rate of 5.0 × 10^−6^.

**Table A6:** DS-CNN results.

Optimizer	Learning Rate	Accuracy	Precision	Recall	F1 Score	Sensitivity	Specificity	AS
Adam	1.0000 × 10^−6^	4.9964 × 10^−1^	5.1862 × 10^−1^	4.9964 × 10^−1^	5.0689 × 10^−1^	3.3333 × 10^−1^	6.2318 × 10^−1^	4.7826 × 10^−1^
	5.0000 × 10^−6^	5.5160 × 10^−1^	5.3563 × 10^−1^	5.5160 × 10^−1^	5.3198 × 10^−1^	2.6598 × 10^−1^	7.6377 × 10^−1^	5.1488 × 10^−1^
	1.0000 × 10^−5^	5.1272 × 10^−1^	5.4502 × 10^−1^	5.1272 × 10^−1^	5.2535 × 10^−1^	4.1091 × 10^−1^	5.8835 × 10^−1^	4.9963 × 10^−1^
	5.0000 × 10^−5^	5.3670 × 10^−1^	5.2086 × 10^−1^	5.3670 × 10^−1^	5.2647 × 10^−1^	3.1117 × 10^−1^	7.0424 × 10^−1^	5.0771 × 10^−1^
	1.0000 × 10^−4^	5.1381 × 10^−1^	5.1635 × 10^−1^	5.1381 × 10^−1^	5.1405 × 10^−1^	3.0861 × 10^−1^	6.6624 × 10^−1^	4.8743 × 10^−1^
SGD	1.0000 × 10^−6^	3.2522 × 10^−1^	4.3285 × 10^−1^	3.2522 × 10^−1^	3.5954 × 10^−1^	2.4979 × 10^−1^	3.8125 × 10^−1^	3.1552 × 10^−1^
	5.0000 × 10^−6^	4.4804 × 10^−1^	5.0521 × 10^−1^	4.4804 × 10^−1^	4.6850 × 10^−1^	3.6573 × 10^−1^	5.0918 × 10^−1^	4.3746 × 10^−1^
	1.0000 × 10^−5^	4.8219 × 10^−1^	5.0488 × 10^−1^	4.8219 × 10^−1^	4.9118 × 10^−1^	3.4101 × 10^−1^	5.8708 × 10^−1^	4.6404 × 10^−1^
	5.0000 × 10^−5^	5.2108 × 10^−1^	5.0838 × 10^−1^	5.2108 × 10^−1^	5.0846 × 10^−1^	2.7366 × 10^−1^	7.0488 × 10^−1^	4.8927 × 10^−1^
	1.0000 × 10^−4^	5.2580 × 10^−1^	5.1234 × 10^−1^	5.2580 × 10^−1^	5.0198 × 10^−1^	2.3359 × 10^−1^	7.4288 × 10^−1^	4.8823 × 10^−1^
Adamax	1.0000 × 10^−6^	4.7711 × 10^−1^	5.2232 × 10^−1^	4.7711 × 10^−1^	4.9520 × 10^−1^	3.6658 × 10^−1^	5.5921 × 10^−1^	4.6290 × 10^−1^
	5.0000 × 10^−6^	5.0363 × 10^−1^	4.9831 × 10^−1^	5.0363 × 10^−1^	5.0071 × 10^−1^	3.1714 × 10^−1^	6.4218 × 10^−1^	4.7966 × 10^−1^
	1.0000 × 10^−5^	5.1890 × 10^−1^	5.4135 × 10^−1^	5.1890 × 10^−1^	5.2757 × 10^−1^	3.9471 × 10^−1^	6.1115 × 10^−1^	5.0293 × 10^−1^
	5.0000 × 10^−5^	5.1417 × 10^−1^	5.3796 × 10^−1^	5.1417 × 10^−1^	5.2337 × 10^−1^	4.1176 × 10^−1^	5.9025 × 10^−1^	5.0101 × 10^−1^
	1.0000 × 10^−4^	5.0363 × 10^−1^	5.2370 × 10^−1^	5.0363 × 10^−1^	5.1203 × 10^−1^	3.8107 × 10^−1^	5.9468 × 10^−1^	4.8788 × 10^−1^
RMSprop	1.0000 × 10^−6^	4.6221 × 10^−1^	5.1987 × 10^−1^	4.6221 × 10^−1^	4.8036 × 10^−1^	4.1603 × 10^−1^	4.9652 × 10^−1^	4.5627 × 10^−1^
	5.0000 × 10^−6^	5.1563 × 10^−1^	5.1527 × 10^−1^	5.1563 × 10^−1^	5.1487 × 10^−1^	3.6488 × 10^−1^	6.2761 × 10^−1^	4.9624 × 10^−1^
	1.0000 × 10^−5^	5.1199 × 10^−1^	5.3299 × 10^−1^	5.1199 × 10^−1^	5.2077 × 10^−1^	3.8875 × 10^−1^	6.0355 × 10^−1^	4.9615 × 10^−1^
	5.0000 × 10^−5^	5.3125 × 10^−1^	5.2037 × 10^−1^	5.3125 × 10^−1^	5.2044 × 10^−1^	2.8389 × 10^−1^	7.1501 × 10^−1^	4.9945 × 10^−1^
	1.0000 × 10^−4^	5.2144 × 10^−1^	5.1892 × 10^−1^	5.2144 × 10^−1^	5.1969 × 10^−1^	3.1287 × 10^−1^	6.7638 × 10^−1^	4.9463 × 10^−1^

## Appendix B. Supplementary Confusion Matrices

Figure A1 provides the confusion matrices for the optimal configuration of the four benchmark models not featured in the main manuscript’s Figure 2. These visualisations offer a complete overview of the error patterns for every architecture evaluated.
